# Estimated Out-of-Pocket Costs for Patients With Common Cancers and Private Insurance

**DOI:** 10.1001/jamanetworkopen.2025.21575

**Published:** 2025-07-21

**Authors:** Liam Rose, Ganesh Rajasekar, Anjali Nambiar, Alexa Pohl, Kathryn J. Ruddy, Katherine Arnow, Manali Patel, Arden M. Morris

**Affiliations:** 1 Stanford University School of Medicine, Palo Alto, California; 2 Department of Veterans Affairs, Palo Alto, California; 3 Department of Surgery, Stanford University School of Medicine, Palo Alto, California; 4 Division of Oncology, Mayo Clinic, Rochester, Minnesota; 5 Division of Oncology, Stanford University School of Medicine, Palo Alto, California; 6 VA Palo Alto Health Care System, Palo Alto, California

## Abstract

**Importance:**

Cancer imposes a substantial economic burden on patients that may be worse in patients with higher-stage disease due to the need for more therapy.

**Objective:**

To investigate the out-of-pocket costs (OOPCs) attributable to the initial treatment of common cancers among privately insured individuals diagnosed at different stages.

**Design, Setting, and Participants:**

This retrospective cohort study used administrative claims data of a large national insurer in the US linked to the Surveillance, Epidemiology, and End Results (SEER) cancer registry to compare OOPCs of individuals diagnosed with breast, colorectal, and lung cancer at different stages with OOPCs of similar individuals without cancer using difference-in-differences methods. Individuals living in the US between 2008 and 2022, aged younger than 65 years, insured through a large national private health insurer, and with 6 or more months of continuous enrollment were included. Data were analyzed from June 2024 through February 2025.

**Exposure:**

The presence or absence of an incident cancer diagnosis and disease stage from the SEER registry diagnosed from 2008 to 2019.

**Main Outcomes and Measures:**

The primary outcome was OOPCs among individuals with breast, colorectal, and lung cancer diagnosed at different disease stages compared with those with no cancer diagnosis.

**Results:**

The cohort consisted of 46 158 patients (mean [SD] age at diagnosis, 46 [12] years; 30 733 female [66.6%]; 2543 Asian [5.5%], 4114 Black [8.9%], 3590 Hispanic [7.8%], and 31 099 White [67.4%]), including 19 656 patients with cancer and 26 502 patients without cancer in the control group. Among patients with cancer, 14 581 patients had breast cancer, 2842 patients had colorectal cancer, and 2233 patients had lung cancer. An incident cancer diagnosis was associated with a mean increase in OOPCs of $592.53 per month (95% CI, $528.01-$627.04 per month) for the 6 months after the diagnosis. Cost monotonically increased with stage at diagnosis (mean OOPC increase range, $462.01 per month [95% CI, $417.92-$506.11 per month] for stage 0 to $719.97 per month [95% CI, $626.11-$813.83 per month] for stage 4).

**Conclusions and Relevance:**

In this study of individuals with private insurance, patients faced high OOPCs after an incident diagnosis of cancer, with patients with more advanced cancer having the highest OOPCs. Further research is needed to determine the clinical and financial effects of increased OOPCs for patients with cancer.

## Key Points

**Question:**

What are the out-of-pocket costs associated with the initial treatment of breast, colorectal, and lung cancer at different stages among privately insured individuals?

**Findings:**

In this cohort study of 46 158 privately insured individuals younger than 65 years, an incident diagnosis of breast, colorectal, or lung cancer was associated with an increase in out-of-pocket costs of $592.53 per month during the 6 months after diagnosis, with costs increasing by stage at diagnosis.

**Meaning:**

This study found that privately insured patients with newly diagnosed cancer, particularly those with more advanced disease, had substantial out-of-pocket costs.

## Teaser

This cohort study investigates the out-of-pocket costs associated with breast, colorectal, and lung cancer among patients younger than 65 years with private insurance.

## Introduction

The increasing costs of cancer care in the US, particularly out-of-pocket costs (OOPCs) borne by patients, have been well documented.^1,2^ This is especially true among Medicare beneficiaries. Patients with Medicare insurance who have an incident cancer diagnosis usually have thousands of dollars of costs annually, and patients without supplemental insurance for Medicare often spend more than half of their annual household income on OOPCs after a diagnosis.^3^ While the Affordable Care Act offered some relief through changes to Medicare Part D, many patients still face large expenditures through inpatient admissions and drug spending.^4,5^ Such financial burden, in turn, has been shown to affect medication adherence.^6,7^ Most patients with cancer are Medicare beneficiaries, explaining the focus on this population; however, with increasing cancer rates among individuals younger than age 65 years who are not yet eligible for Medicare, novel data sources from the private sector are needed to understand OOPCs among this younger demographic.^8^

Several obstacles have made estimating OOPCs associated with specific cancer diagnoses difficult in the privately insured US adult population. Survey-based datasets typically used to address consumer health care costs (eg, the Medical Expenditure Panel Survey) lack the sample size needed to stratify by site (ie, type) and stage^9^ in addition to systematically underestimating costs.^10^ Claims databases, such as MarketScan and OptumLabs Data Warehouse, provide adequate sample size and accurate cost data. However, staging information is inconsistently available in claims databases and, when present, lacks the rigor of record review present in population cancer registries (eg, the Surveillance, Epidemiology, and End Results [SEER] registry). A link between claims and clinical data is necessary to identify patient populations most at risk for high OOPCs. While the linkage between Medicare claims records and SEER data has been explored, such linkages have remained elusive for the privately insured population.

This study aimed to fill this gap by leveraging a novel linkage between SEER and the largest private insurer in the US. These data combine claims records from the OptumLabs database with SEER to accurately capture cancer-related variables, including site and stage at diagnosis, along with cost variables specific to an individual’s insurance plan. This dataset allows isolation of costs directly attributable to the cancer diagnosis from baseline medical expenditures. We used these data to compare OOPCs for patients with cancer before and after their diagnosis with those of patients without cancer using a difference-in-differences (DiD) strategy to help avoid confounding factors in individuals’ medical expenditures over time.

## Methods

The Stanford University Institutional Review Board approved this cohort study and ruled it exempt from informed consent. This sutdy followed the Strengthening the Reporting of Observational Studies in Epidemiology (STROBE) reporting guideline. We used the OptumLabs-SEER registry to identify privately insured patients with new cancer diagnoses. This dataset links population-based clinical data recorded by the SEER program to medical claims records for individuals enrolled in Optum health insurance plans. Breast, lung, and colorectal cancer cases were identified based on *International Classification of Diseases for Oncology, Third Revision* (*ICD-O-3*) SEER site and histology codes (eTable 4 in Supplement 1) diagnosed from January 1, 2008, to December 31, 2019, among patients ages 18 to 64 years at the time of diagnosis. Cancer stage was identified by the American Joint Committee on Cancer (AJCC) *Cancer Staging Manual* 6th edition for diagnoses from 2008-2010, AJCC 7th edition from 2010-2015, Derived SEER Stage Group from 2016-2017, and Derived Extent of Disease Stage Group from 2018-2019. Diagnosis day was not in these data due to privacy concerns, so all diagnosis dates were set at the first day of the corresponding month and year of diagnosis.

Among patients with cancer, race and ethnicity information was derived from Optum SEER registry data. This information was abstracted from patient medical records using North American Association of Central Cancer Registries criteria and consolidated into 6 mutually exclusive categories. Among patients in the control group, race and ethnicity information was derived from Optum payer profile data. Race and ethnicity were included as part of the demographics of the study population.

Medical claims were then extracted for each patient from January 1, 2007, to January 1, 2021, and applied for the time span of interest. Claims were extracted on October 29, 2024. Elixhauser scores were calculated from diagnoses on claims.

Patients were excluded if they were aged 65 years or older, enrolled in a Medicare Advantage plan at any time during their enrollment in Optum, had no associated medical claims, or were missing cancer stage. All patients with continuous coverage in Optum data less than 6 months before or after the diagnosis date were excluded, although we conducted a sensitivity analysis of this parameter (eFigure 2 in Supplement 1). Patients with stage 0 lung cancer or colorectal cancer were excluded due to low sample sizes and lack of clinical significance. Claims with negative OOPCs were also excluded because they resulted from adjustments made outside of regular claim-processing rules or from incorrect charges to patients that were later reversed. Flowcharts of exclusions for each group of patients with cancer and the control group are in eFigure 3 in Supplement 1.

We randomly sampled individuals without cancer as a comparison group by assigning random pseudodiagnosis dates to all individuals without cancer in the time span of interest. We then excluded individuals without 6 months of continuous coverage before and after these dates. We further excluded individuals who had a cancer diagnosis any time before their pseudodiagnosis date or up to 2 years after that date, were younger than age 18 years or older than age 65 years, were enrolled in any Medicare Advantage plan, or had no associated medical claims in OptumLabs’ database. Further details regarding the control cohort are listed in the eMethods in Supplement 1.

Comparing OOPCs for patients with cancer before and after a diagnosis may be subject to confounding due to temporal trends in costs, baseline medical expenditures, and OOPCs incurred before the official diagnosis date listed in the cancer registry. To avoid these issues, we implemented a staggered DiD approach following Gardner et al.^11^ This is a 2-stage DiD approach that allows for treatment effect heterogeneity and variation in treatment timing. Specifically, we compared OOPCs for individuals with incident cancer diagnoses vs OOPCs of individuals without a cancer diagnosis before and after the diagnosis date. Regressions included controls for age, race and ethnicity, education, and income, all of which were collected by Optum. Standard errors were clustered at the relative month-from-diagnosis date or pseudodiagnosis date level. We used event study figures to demonstrate evidence of parallel trends assumptions along with estimating the average treatment effect for OOPCs in the 6 months after the diagnosis (defined as month 0 and the proceeding 6 months). We further replicated this estimation by stage at the time of diagnosis. Finally, as noted previously, we repeated the analysis for different levels of continuous insurance coverage, ranging from 3 months before and after the diagnosis date to 12 months before and after the diagnosis date.

OOPCs were identified from medical claims and aggregated by month, relative to the month and year of diagnosis. This included any copay, coinsurance, or deductible that was associated with any claim in that month. Patients who were enrolled but had no medical claims in a given month were assigned OOPCs of $0 for that month. High-deductible health plans were defined based on a patient insurance plan having an individual deductible greater than or equal to $1400 per year or a family deductible for all members on that plan of $2800 per year or greater. All dollar amounts were inflation adjusted to 2024 dollars. Analyses were conducted in R statistical software version 4.4.1 (R Project for Statistical Computing) and SAS statistical software version 9.4 (SAS Institute). Data were analyzed from June 2024 through February 2025.

## Results

There were 46 158 patients in our cohort (mean [SD] age at diagnosis, 46 [12] years; 30 733 female [66.6%]; 2543 Asian [5.5%], 4114 Black [8.9%], 3590 Hispanic [7.8%], and 31 099 White [67.4%]), including 19 656 patients with cancer (42.6%) and 26 502 patients without cancer (57.4%) in the control group (Table 1). Most patients with cancer (14 581 patients [74.1%]) had breast cancer, while 2842 patients (14.5%) had colorectal cancer and 2233 patients (11.4%) had lung cancer. Patients with cancer were older and had a higher comorbidity burden than patients in the control group. Overall, 19 865 patients (43.0%) had some college education and 11 031 patients (23.9%) had incomes between $75 000 and $124 999 per year. Summary statistics by stage at diagnosis for each type of cancer are available in eTables 1 to 3 in Supplement 1.

**Table 1.** Patient Characteristics

Characteristic	Patients, No. (%)					*P* value
	All (N = 46 158)	Breast cancer (n = 14 581)	Colorectal cancer (n = 2842)	Lung cancer (n = 2233)	Control (n = 26 502)	
Age at time of diagnosis, mean (SD), y	46 (12)	52 (8)	53 (8)	57 (6)	42 (13)	<.001
Elixhauser Comorbidity Index score, mean (SD)	1 (1)	1 (1)	1 (2)	2 (2)	1 (1)	<.001
Sex						
Female	30 733 (66.6)	14 496 (99.4)	1268 (44.6)	1174 (52.6)	13 795 (52.1)	<.001
Male	15 425 (33.4)	85 (0.6)	1574 (55.4)	1059 (47.4)	12 707 (47.9)	
Race and ethnicity						
Asian	2543 (5.5)	1035 (7.1)	162 (5.7)	<100 (<5)	1256 (4.7)	<.001
Hispanic	3590 (7.8)	938 (6.4)	185 (6.5)	<100 (<5)	2376 (9.0)	
Non-Hispanic Black	4114 (8.9)	1557 (10.7)	360 (12.7)	203 (9.1)	1994 (7.5)	
Non-Hispanic White−	31 099 (67.4)	10947 (75.1)	2113 (74.3)	1845 (82.6)	16194 (61.1)	
Other^a^	4812 (10.4)	104 (0.7)	22 (0.8)	<10 (<0.1)	4682 (17.7)	
Education						
<12th Grade	273 (0.6)	58 (0.4)	13 (0.5)	13 (0.6)	189 (0.7)	<.001
High school diploma	9860 (21.4)	2764 (19.0)	697 (24.5)	584 (26.2)	5815 (21.9)	
Some college	19 865 (43.0)	6354 (43.6)	1171 (41.2)	857 (38.4)	11483 (43.3)	
≥Bachelor’s degree	9653 (20.9)	3771 (25.9)	548 (19.3)	309 (13.8)	5025 (19.0)	
Unknown	6507 (14.1)	1634 (11.2)	413 (14.5)	470 (21.0)	3990 (15.1)	
Annual household income, $						
<40 000	4843 (10.5)	1432 (9.8)	313 (11.0)	246 (11.0)	2852 (10.8)	<.001
40 000-74 999	8287 (18.0)	2340 (16.0)	500 (17.6)	390 (17.5)	5057 (19.1)	
75 000-124 999	11 031 (23.9)	3572 (24.5)	722 (25.4)	508 (22.7)	6229 (23.5)	
125 000-199 999	7293 (15.8)	2660 (18.2)	435 (15.3)	277 (12.4)	3921 (14.8)	
≥200 000	5968 (12.9)	2471 (16.9)	344 (12.1)	207 (9.3)	2946 (11.1)	
Unknown	8736 (18.9)	2106 (14.4)	528 (18.6)	605 (27.1)	5497 (20.7)	
High-deductible health plan	14 686 (31.8)	4274 (29.3)	923 (32.5)	630 (28.2)	8859 (33.4)	<.001

^a^ Other includes categories of other and unknown race and ethnicity among patients with cancer and unknown race and ethnicity among patients in the control group.

**Figure 1.**
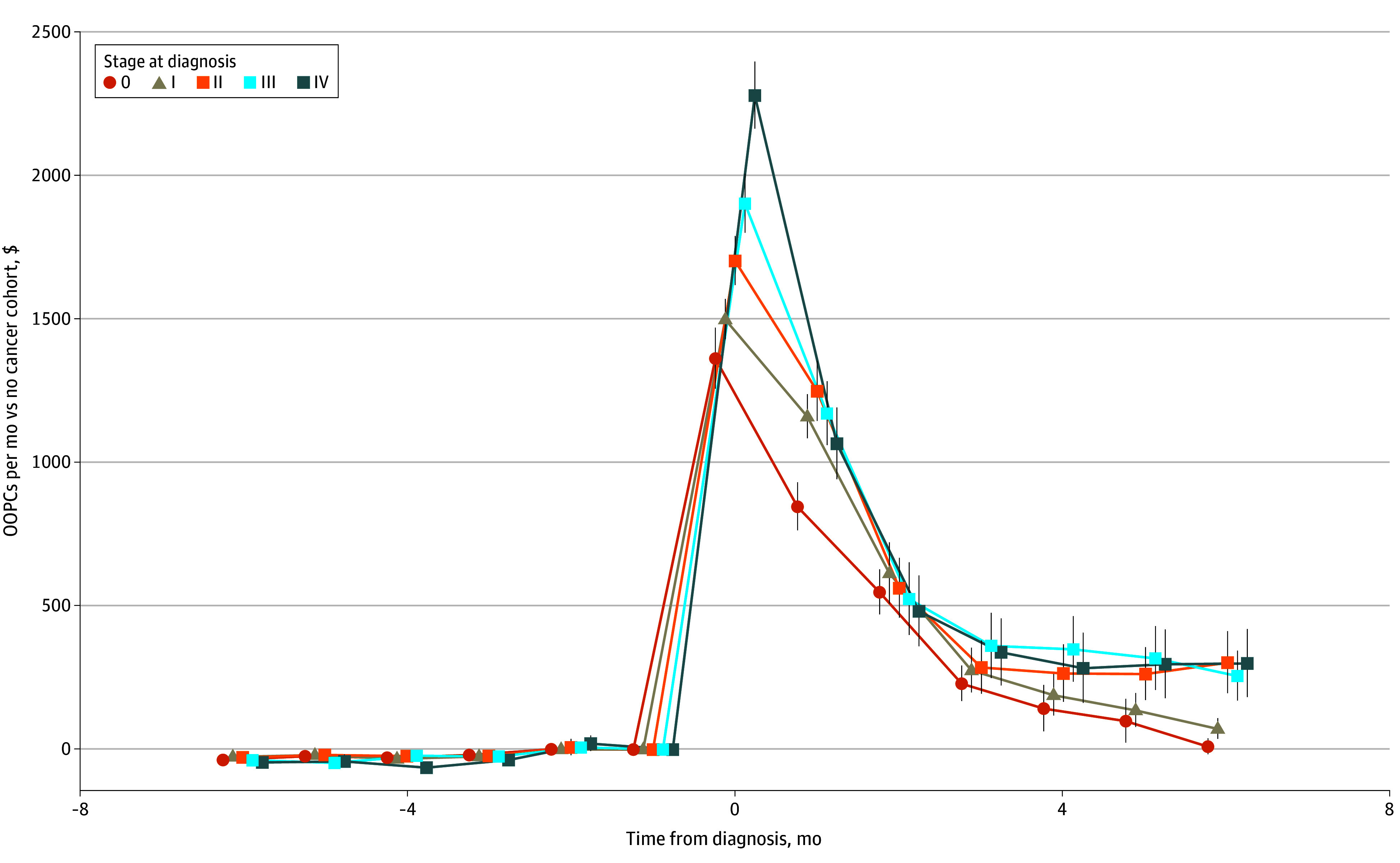
Event Study Figure of Difference in Costs Between Cancer and Control Groups The difference in out-of-pocket costs (OOPCs) is shown by month for patients with a cancer diagnosis compared with patients without cancer (control group). The x-axis is listed in terms of months away from the diagnosis or pseudodiagnosis date, centered at 0, with points offset for visibility.

Among patients grouped by stage at diagnosis, we found no significant difference in monthly OOPCs before the diagnosis (Figure 1). However, we observed a spike in OOPCs in the month of the diagnosis, and costs remained higher for patients with cancer than those without cancer for 6 months after the diagnosis month. OOPCs were higher for patients with more advanced stage at diagnosis. DiD estimates for the 6-month period before and after the diagnosis date are displayed in Table 2. Patients with cancer had costs that were a mean of $592.53 per month (95% CI, $528.01-$627.04 per month) higher than those for patients without cancer in the 6 months after the diagnosis date. The mean OOPC increase ranged from $462.01 per month (95% CI, $417.92-$506.11 per month) for patients with stage 0 cancer to $719.97 per month (95% CI, $626.11-$813.83 per month) for patients with stage 4 cancer. See eTable 5 in Supplement 1 for estimates for these differences by month.

**Table 2.** Estimated Change in OOPCs After Cancer Diagnosis

Cancer stage	Observations, No.	Patients with cancer, No.	Estimated DiD change in OOPCs, mean (95% CI), $^a^
All	601 653	19 774	592.53 (528.01-657.04)
0	389 025	3423	462.01 (417.92-506.11)
I	440 518	7347	563.05 (503.51-622.59)
II	401 908	4409	660.70 (581.38-740.01)
III	378 443	2606	696.52 (609.28-783.77)
IV	370 435	1989	719.97 (626.11-813.83)

Abbreviations: DiD, difference-in-differences; OOPC, out-of-pocket cost.

^a^ Cancers include breast, colorectal, or lung cancer. DiD estimates of the change in OOPCs are given relative to the noncancer cohort, 2008-2019. Estimates are shown for all cancer diagnoses and by stage at diagnosis from the Surveillance, Epidemiology, and End Results (SEER) registry.

**Figure 2.**
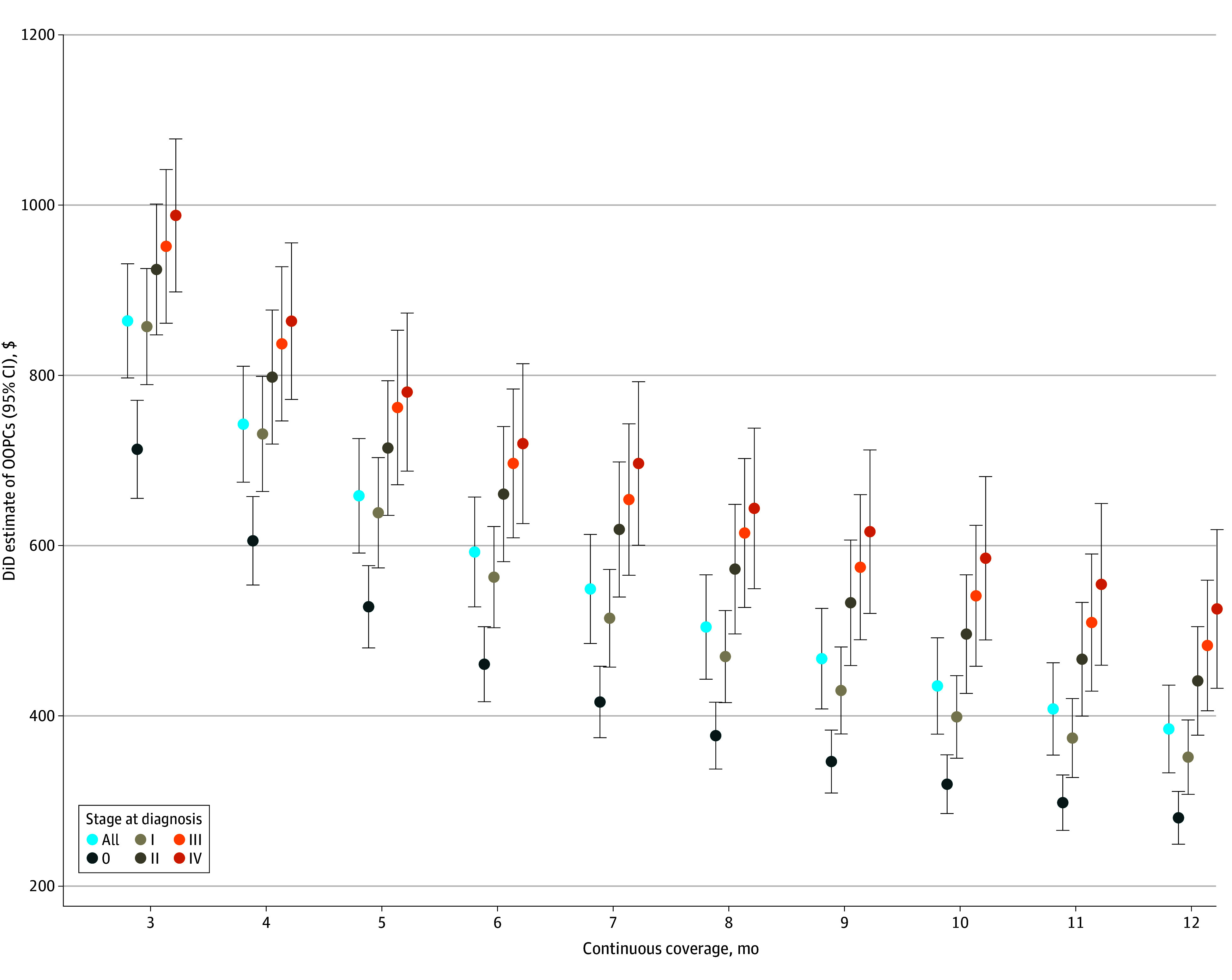
Sensitivity of Cost Estimates to Continuous Insurance Coverage Requirement Difference-in-differences (DiD) estimates of out-of-pocket costs (OOPCs) for each minimum value of continuous coverage before and after the diagnosis or pseudodiagnosis date are listed along the x-axis, from 3 months to 12 months.

The sensitivity of these DiD estimates to a range of continuous insurance coverage requirements, from 3 months before and after the diagnosis date to 12 months before and after the diagnosis date, is shown in Figure 2. Each set of estimates along the x-axis of the figure represents a modified sample from our main estimates except for the set of estimates representing 6 months of continuous coverage. The figure shows that increasing the continuous coverage requirement was associated with lower estimates of the difference in OOPCs between patients with cancer and those without cancer, with the mean monthly difference being the smallest with 12 months of continuous coverage required before and after diagnosis ($384.41 per month; 95% CI, $332.87-$435.96 per month) (eTable 6 in Supplement 1).

## Discussion

This retrospective cohort study of patients in a cancer registry aged younger than 65 years linked with private insurance claims found a significant increase in OOPCs among patients with cancer relative to those without cancer, starting before their diagnosis date. We found a mean monthly difference of $592.53; including the month of diagnosis and 6 months after, this would indicate cumulative additional OOPCs of $4144.71. This difference, driven by the onset of cancer diagnosis and its associated treatment, underscores the financial burden of cancer care on patients with insurance who are not yet eligible for Medicare. More importantly, we found that there were significant differences in OOPCs for patients diagnosed at later stages. This result seems straightforward; later-stage disease is associated with more intensive workup and treatment that can drive higher medical expenditures. However, this result has not previously been empirically demonstrated, to our knowledge. Our findings provide quantitative evidence that even with private insurance, OOPCs were higher in the month of diagnosis and in the months after diagnoses compared with costs for a control group.^12^ Moreover, patients whose disease was diagnosed at a more advanced stage had the highest OOPCs across a range of common cancers.

Among cancer survivors, 1 in 2 individuals experiences financial toxicity, or financial burdens that substantially disrupt their quality of life and access to health care, after being diagnosed with cancer.^13^ In addition to its negative effects on health-related quality of life, financial toxicity contributes to worse cancer outcomes as patients forgo recommended cancer treatments, cancer survivorship care, and treatment of other chronic medical conditions.^14^ Our understanding of this phenomenon among individuals enrolled in Medicare is relatively thorough; cancer registry–linked Medicare data have been available to researchers since 2001, and Medicare finances and cost sharing are a near-constant public policy debate. However, our understanding of financial toxicity outside of the Medicare population is relatively poor. Along with data sparsity, there is vast variation in health insurance plans available to individuals and households, contributing to substantial heterogeneity in what costs a patient may have after diagnosis. It has also been shown that switching to a high-deductible plan greatly increases OOPCs for patients with cancer and may delay treatment.^15,16^ Perhaps most importantly, health insurance coverage is generally linked with employment for the working age population, with more than 60% of working age adults relying on employer-sponsored insurance.^17^ This creates significant uncertainty in understanding the full scope of acute and cumulative financial toxicity for patients with cancer who do not have Medicare and who lose or end their employment, making it a critical area for future research and policy intervention.

### Limitations

Our study has several limitations. To avoid conflating baseline medical expenditures to OOPs among patients with cancer before and after a diagnosis, this study used a DiD approach. The primary assumption of any DiD analysis is that the treated cohort would evolve similarly to the comparison cohort in the absence of any intervention. Our finding that the difference in OOPCs between the cancer and noncancer groups was negligible in the months before the cancer diagnosis date provides evidence of this (Figure 1). However, our analysis is also subject to another potential confounder: differential attrition. Individuals with cancer may drop their insurance coverage at differing rates than those without cancer due to mortality, loss of employment, or a desire to change insurance coverage. While our use of the 2-stage DiD approach allows for treatment effect heterogeneity, reasons for dropping insurance are not available in these data, and differential attrition may have biased our estimates upward if a significant portion of the cancer group dropped their insurance coverage shortly after receiving an incident diagnosis. We therefore explored how the sensitivity of our estimates changed with differing thresholds for continuous coverage. Patients with stage IV cancer were by far the most likely to drop out. However, this limitation is unavoidable in the absence of all-payer claims data. We attempted to mitigate this limitation, as noted in Figure 2, by showing that our main estimate with the continuous coverage requirement set at least 6 months was in the middle of the estimates of the mean monthly OOPCs. An additional sensitivity analysis of attrition showed that the noncancer group was consistently more likely to drop out from our sample than the cancer group (eFigure 1 in Supplement 1). This is consistent with previous research on job and health plan lock.^18^ This was again shown stratified by stage at diagnosis (eFigure 2 in Supplement 1). This indicated that individuals diagnosed at stage IV were by far the most likely patients to drop from the sample.

While the direct medical OOPCs that we analyzed in this study are the largest driver of expenditures for patients with cancer in treatment, it has been well documented that patients have many other costs, such as travel and lost labor income.^1^ However, such data are not readily available for claims-based studies and do not fall within the accepted definition of OOPCs.

## Conclusions

In this cohort study, patients with private insurance were found to have high OOPCs after an incident diagnosis of cancer, and those with the most advanced cancer had the highest OOPCs. The variability in OOPCs based on cancer stage underscores the need for policies such as paid sick leave, that address both insurance continuity and financial assistance, especially for patients with more advanced cancer.

## Notes

Supplement 1. **eTable 1.** Characteristics of Patients With Breast Cancer by Stage at Diagnosis **eTable 2.** Characteristics of Patients With Lung Cancer by Stage at Diagnosis **eTable 3.** Characteristics of Patients With Colorectal Cancer by Stage at Diagnosis **eMethods.** Sampling Method for Control Group **eTable 4.** *ICD-O-3* and Histology Codes Used to Identify Patients With Cancer **eTable 5.** Difference in OOPCs by Month Between Patients With and Without Cancer by Stage at Diagnosis **eTable 6.** Sensitivity of Difference-in-Difference Estimate of OOPCs to Continuous Insurance Coverage Requirement **eFigure 1.** Attrition From Sample by Cancer Status **eFigure 2.** Attrition From Sample by Stage at Diagnosis **eFigure 3.** Flowchart of Exclusions

Supplement 2.
Data Sharing Statement
