# Supplementary material for: Estimated Out-of-Pocket Costs for Patients With Common Cancers and Private Insurance
Source: JAMA Netw Open. 2025 Jul 21;8(7):e2521575. doi: 10.1001/jamanetworkopen.2025.21575 (PMC12281234; doi:10.1001/jamanetworkopen.2025.21575)
Supplement: Supplement 1. — eTable 1. Characteristics of Patients With Breast Cancer by Stage at Diagnosis eTable 2. Characteristics of Patients With Lung Cancer by Stage at Diagnosis eTable 3. Characteristics of Patients With Colorectal Cancer by Stage at Diagnosis eMethods. Sampling Method for Control Group eTable 4. ICD-O-3 and Histology Codes Used to Identify Patients With Cancer eTable 5. Difference in OOPCs by Month Between Patients With and Without Cancer by Stage at Diagnosis eTable 6. Sensitivity of Difference-in-Difference Estimate of OOPCs to Continuous Insurance Coverage Requirement eFigure 1. Attrition From Sample by Cancer Status eFigure 2. Attrition From Sample by Stage at Diagnosis eFigure 3. Flowchart of Exclusions [file jamanetwopen-e2521575-s001.pdf]

Supplemental Online Content

Rose L, Rajasekar G, Nambiar A, et al. Estimated Out-of-Pocket Costs for Patients With Common Cancers and Private Insurance. *JAMA Netw Open*. 2025;8(7):e2521575. doi:10.1001/jamanetworkopen.2025.21575

eTable 1. Characteristics of Patients With Breast Cancer by Stage at Diagnosis

eTable 2. Characteristics of Patients With Lung Cancer by Stage at Diagnosis

eTable 3. Characteristics of Patients With Colorectal Cancer by Stage at Diagnosis

eMethods. Sampling Method for Control Group

eTable 4. *ICD-O-3* and Histology Codes Used to Identify Patients With Cancer

eTable 5. Difference in OOPCs by Month Between Patients With and Without Cancer by Stage at Diagnosis

eTable 6. Sensitivity of Difference-in-Difference Estimate of OOPCs to Continuous Insurance Coverage Requirement

eFigure 1. Attrition From Sample by Cancer Status

eFigure 2. Attrition From Sample by Stage at Diagnosis

eFigure 3. Flowchart of Exclusions

This supplemental material has been provided by the authors to give readers additional information about their work.

© 2025 Rose L et al. *JAMA Network Open*.

eTable 1. Characteristics of Breast Cancer Patients by Stage at Diagnosis

	Stage 0 (n= 3412)	Stage 1 (n=6162)	Stage 2 (n=3536)	Stage 3 (n=1087)	Stage 4 (n=384)	p-value
**Age at time of Diagnosis, Mean (SD)**	52 (8)	53 (8)	51 (9)	50 (9)	51 (9)	<0.001
**Elixhauser Comorbidity Index, Mean (SD)**	1 (1)	1 (1)	1 (1)	1 (1)	1 (1)	<0.001
**Female, n (%)**	3398 (100)	6128 (99)	3513 (99)	1078 (99)	379 (99)	0.15
**Race/Ethnicity, n (%)**						<0.001
**Asian**	253 (7)	422 (7)	252 (7)	<100 (<10)	<50 (<10)	
**Hispanic**	206 (6)	380 (6)	251 (7)	78 (7)	23 (6)	
**Non-Hispanic Black**	362 (11)	540 (9)	430 (12)	167 (15)	58 (15)	
**Non-Hispanic White**	2561 (75)	4774 (77)	2580 (73)	763 (70)	269 (70)	
**Other***	30 (1)	46 (1)	23 (1)	<5 (**)	<5 (**)	
**Education, n (%)**						<0.001
**Less than 12th**	15 (0)	19 (0)	19 (1)	<10 (**)	<5 (**)	
**High School Diploma**	606 (18)	1116 (18)	710 (20)	248 (23)	<100 (<30)	
**Some College**	1402 (41)	2723 (44)	1588 (45)	489 (45)	152 (40)	
**Bachelors Degree or More**	1000 (29)	1674 (27)	827 (23)	199 (18)	71 (18)	
**Unknown**	389 (11)	630 (10)	392 (11)	<200 (<20)	77 (20)	
**HH Income, n(%)**						<0.001
**<$40,000**	297 (9)	550 (9)	411 (12)	135 (12)	39 (10)	
**$40,000-$74,999**	505 (15)	948 (15)	617 (17)	205 (19)	65 (17)	
**$75,000-$124,999**	801 (23)	1539 (25)	891 (25)	254 (23)	87 (23)	
**$125,000-$199,999**	649 (19)	1166 (19)	602 (17)	181 (17)	62 (16)	
**$200,000+**	666 (20)	1127 (18)	515 (15)	128 (12)	35 (9)	
**Unknown**	494 (14)	832 (14)	500 (14)	184 (17)	96 (25)	
**HDHP, n (%)**	900 (26)	1883 (31)	1057 (30)	304 (28)	130 (34)	<0.001
**Total OOPC, Mean (SD) ** **	2393 (3145)	2422 (2179)	2418 (2098)	2468 (2350)	2967 (2451)	<0.001
**One-Month OOPC, Mean (SD) *****	109 (398)	108 (427)	93 (366)	73 (250)	108 (534)	0.04
**Average Monthly OOPC, Mean (SD) *******	342 (449)	346 (311)	345 (300)	353 (336)	424 (350)	0.001

* Includes "Other" and "Unknown" Race/Ethnicity among Cancer Patients and "Unknown" Patients for Control Patients
** Total OOPC aggregated from 6 Months prior to diagnosis to diagnosis
*** Average Mean OOPC at 6 Months prior to diagnosis

© 2025 Rose L et al. *JAMA Network Open*.

**** OOPC Monthly Average ($ per month) from 6 Months prior to diagnosis to date of diagnosis

© 2025 Rose L et al. *JAMA Network Open*.

eTable 2. Characteristics of Lung Cancer Patients by Stage at Diagnosis

	Stage 1 (n=558)	Stage 2 (n=194)	Stage 3 (n=520)	Stage 4 (n=961)	**p-value**
**Age at time of Diagnosis, Mean (SD)**	57 (7)	57 (6)	57 (6)	56 (6)	0.0778
**Elixhauser Comorbidity Index, Mean (SD)**	2 (2)	2 (2)	2 (2)	2 (2)	<0.001
**Female, n (%)**	337 (60)	90 (46)	269 (52)	478 (50)	0.0003
**Race/Ethnicity, n (%)**					0.08
**Asian**	<20 (<5)	<10 (**)	<20 (<5)	<60 (<10)	
**Hispanic**	20 (4)	<10(**)	21 (4)	43 (4)	
**Non-Hispanic Black**	49 (9)	19 (10)	37 (7)	98 (10)	
**Non-Hispanic White**	472 (85)	162 (84)	447 (86)	764 (80)	
**Other***	<5 (**)	<5 (**)	<5 (**)	<5 (**)	
**Education, n(%)**					0.0002
**Less than 12th**	<5 (**)	<5 (**)	<5 (**)	<5 (**)	
**High School Diploma**	140 (25)	57 (29)	145 (28)	242 (25)	
**Some College**	230 (41)	70 (36)	197 (38)	360 (37)	
**Bachelors Degree or More**	99 (18)	35 (18)	59 (11)	<120 (<15)	
**Unknown**	<90 (<20)	<40 (<20)	<150 (<25)	238 (25)	
**HH Income, n (%)**					<0.001
**<$40,000**	66 (12)	28 (14)	62 (12)	90 (9)	
**$40,000-$74,999**	94 (17)	37 (19)	90 (17)	169 (18)	
**$75,000-$124,999**	150 (27)	45 (23)	118 (23)	195 (20)	
**$125,000-$199,999**	88 (16)	21 (11)	59 (11)	109 (11)	
**$200,000+**	58 (10)	21 (11)	36 (7)	92 (10)	
**Unknown**	102 (18)	42 (22)	155 (30)	306 (32)	
**HDHP, n (%)**	157 (28)	61 (31)	145 (28)	267 (28)	0.77
**Total OOPC, Mean (SD) ****	3125 (2639)	3569 (5660)	3025 (2298)	3406 (2957)	0.04
**One-Month OOPC, Mean (SD) *****	165 (545)	151 (770)	115 (424)	103 (399)	0.09
**Average Monthly OOPC, Mean (SD) *******	446 (377)	510 (809)	432 (328)	487 (422)	0.04

* Includes "Other" and "Unknown" Race/Ethnicity among Cancer Patients and "Unknown" Patients for Control Patients
** Total OOPC aggregated from 6 Months prior to diagnosis to diagnosis
*** Average Mean OOPC at 6 Months prior to diagnosis

© 2025 Rose L et al. *JAMA Network Open*.

**** OOPC Monthly Average ($ per month) from 6 Months prior to diagnosis to date of diagnosis

© 2025 Rose L et al. *JAMA Network Open*.

eTable 3. Characteristics of Colorectal Cancer Patients by Stage at Diagnosis

	Stage 1 (n=595)	Stage 2 (n=651)	Stage 3 (n=976)	Stage 4 (n=620)	p-value
**Age at time of Diagnosis, Mean (SD)**	54 (7)	54 (8)	52 (8)	52 (8)	<0.001
**Elixhauser Comorbidity Index, Mean (SD)**	1 (2)	1 (2)	1 (2)	1 (2)	0.002
**Female, n (%)**	280 (47)	287 (44)	428 (44)	273 (44)	0.51
**Race/Ethnicity, n (%)**					0.01
**Asian**	35 (6)	<40 (<10)	<60 (<10)	<40 (<10)	
**Hispanic**	<50 (<10)	49 (8)	58 (6)	39 (6)	
**Non-Hispanic Black**	99 (17)	85 (13)	101 (10)	75 (12)	
**Non-Hispanic White**	413 (69)	474 (73)	754 (77)	472 (76)	
**Other***	<10 (**)	<5 (**)	<5 (**)	<5 (**)	
**Education, n(%)**					<0.001
**Less than 12th**	<5 (**)	<5 (**)	<5 (**)	<5 (**)	
**High School Diploma**	146 (25)	178 (27)	241 (25)	132 (21)	
**Some College**	244 (41)	277 (43)	404 (41)	246 (40)	
**Bachelors Degree or More**	141 (24)	117 (18)	189 (19)	<110 (<20)	
**Unknown**	<100 (<15)	<80 (<15)	<150 (<15)	137 (22)	
**HH Income, n(%)**					<0.001
**<$40,000**	67 (11)	90 (14)	104 (11)	52 (8)	
**$40,000-$74,999**	104 (17)	116 (18)	173 (18)	107 (17)	
**$75,000-$124,999**	155 (26)	190 (29)	242 (25)	135 (22)	
**$125,000-$199,999**	94 (16)	98 (15)	148 (15)	95 (15)	
**$200,000+**	96 (16)	56 (9)	132 (14)	60 (10)	
**Unknown**	79 (13)	101 (16)	177 (18)	171 (28)	
**HDHP, n(%)**	200 (34)	195 (30)	318 (33)	210 (34)	0.42
**Total OOPC,Mean (SD) ****	2563 (2417)	3106 (2558)	3119 (2805)	3330 (2770)	<0.001
**One-Month OOPC Mean (SD) *****	101 (423)	89 (331)	100 (481)	83 (480)	0.85
**Average Monthly OOPC, Mean (SD) *******	366 (345)	444 (365)	446 (401)	476 (396)	<0.001

* Includes “Other” and “Unknown” Race/Ethnicity among Cancer Patients and "Unknown" Patients for Control Patients
** Total OOPC aggregated from 6 Months prior to diagnosis to diagnosis
*** Average Mean OOPC at 6 Months prior to diagnosis
**** OOPC Monthly Average ($ per month) from 6 Months prior to diagnosis to date of diagnosis

© 2025 Rose L et al. *JAMA Network Open*.

eMethods. Sampling Method for Control Group

To sample the control group from the full Optum Labs Data Warehouse data, we first split the cancer cohorts into separate datasets by year of diagnosis, henceforth called “yearly datasets” (e.g. all people diagnosed in 2008, 2009, etc.).

Then, for each yearly dataset, we sampled 8,000 patients from the Optum continuous medical coverage dataset from 2008-2024 with replacement who had coverage during the corresponding year of diagnosis. This resulted in 96,000 observations representing diagnoses from 2008 to 2019. We then generated a pseudo-month of diagnosis based on the probability of the month of diagnosis among the cancer patients. For example, if 12% of cancer patients were diagnosed in June 2015, there was a 12% chance that a control could be “diagnosed” in June among the 2015 yearly dataset. All control patients were then assigned the same diagnosis day of 1 due to the omission of this data from Optum Labs. If a patient was randomly sampled in more than one yearly dataset, the earliest pseudo-diagnosis date was kept for each person. As a result, 66 diagnoses were removed, resulting in an initial cohort of 95,934 control patients. Our objective when doing this was to maximize our initial control cohort while also performing our analyses within the computational limitations of Optum’s servers.

© 2025 Rose L et al. *JAMA Network Open*.

eTable 4. ICD-O-3 and Histology Codes used to Identify Cancer Patients

Cancer Type	ICD-O-3 Code(s)	Histology Code(s)
Breast	C500-C506, C508-C509	800,801,802,805,814,821,823,825,826,832,844,847,848,849,850,851,852,853,854,855,856,857,898
Lung	C340-C343, C348-C349	800,801,802,803,804,805,807,808,809,810,812,814,820,823,824,825,826,831,832,843,848,849,856,857,880,881
Colorectal	C209	800,801,802,814,822,823,824,825,826,844,848,849
	C180, C182-C189, C199	800,801,802,804,814,822,823,825,848,849,851

© 2025 Rose L et al. *JAMA Network Open*.

eTable 5. Difference in OOPC by Month Between Cancer and Non-Cancer Patients by Stage at Diagnosis

	**SEER Stage at Diagnosis (DiD Estimate ($) 95% CI)**				
**Months from Diagnosis Date**	**Stage 0**	**Stage 1**	**Stage 2**	**Stage 3**	**Stage 4**
**- 6**	--36.30 (-47.64, -24.96)	-25.32 (-35.26, -15.38)	-27.38 (-39.07, -15.69)	-37.28 (-51.46, -23.11)	-44.74 (-63.41, -26.06)
**- 5**	-23.43 (-36.98, -9.87)	-19.50 (-29.32, -9.68)	-18.82 (-31.48, -6.16)	-46.37 (-58.47, -34.26)	-41.31 (-63.79, -18.84)
**- 4**	-28.41 (-41.67, -15.16)	-32.38 (-41.96, -22.80)	-22.83 (-34.9, -10.76)	-21.83 (-40.11, -3.55)	-63.01 (-78.90, -47.12)
**- 3**	-19.22 (-32.93, -5.51)	-23.98 (-33.86, -14.09)	-22.25 (-33.23, -11.27)	-23.94 (-37.35, -10.52)	-36.37 (-56.16, -16.58)
**- 2**	0.85 (-13.24,14.94)	0.59 (-9.86,11.04)	7.32 (-21.03,35.66)	7.21 (-9.28,23.69)	20.58 (-6.42,47.59)
**- 1**	N/A	N/A	N/A	N/A	N/A
**0**	1360.80 (1254.45,1467.15)	1496.8 (1426.70,1566.91)	1700.63 (1615.10,1786.17)	1899.90 (1797.33,2002.47)	2276.37 (2159.62,2393.12)
**1**	845.04 (761.35,928.74)	1158.64 (1081.77,1235.5)	1247.19 (1142.42,1351.96)	1169.33 (1057.96,1280.71)	1064.35 (939.41,1189.29)
**2**	547.48 (469.16,625.79)	613.18 (506.65,719.70)	561.45 (457.20,665.69)	523.77 (397.39,650.16)	481.31 (358.17,604.45)
**3**	229.26 (167.68,290.84)	275.40 (197.61,353.20)	285.65 (193.13,378.16)	361.01 (247.50,474.52)	338.34 (221.88,454.80)
**4**	142.53 (61.78,223.27)	189.14 (116.95,261.33)	264.72 (164.78,364.65)	348.68 (234.54,462.83)	282.94 (160.64,405.23)
**5**	98.88 (22.73,175.03)	136.30 (77.44,195.16)	262.80 (170.85,354.75)	317.10 (205.84,428.37)	296.86 (177.23,416.49)
**6**	10.10 (-17.43,37.63)	71.87 (35.99,107.74)	302.44 (194.55,410.34)	255.84 (169.23,342.45)	299.61 (181.36,417.86)

© 2025 Rose L et al. *JAMA Network Open*.

eTable 6. Sensitivity of Difference-in-Difference Estimate of OOPC to Continuous Insurance Coverage Requirement

	SEER Stage at Diagnosis (DiD Estimate ($) 95% CI)					
**Months of Continuous Coverage**	**All**	**Stage 0**	**Stage 1**	**Stage 2**	**Stage 3**	**Stage 4**
**3**	863.32 (796.29, 930.36)	712.62 (655.09, 770.14)	856.61 (788.48, 924.75)	923.64 (846.96, 1000.33)	950.81 (860.6, 1041.02)	987.09 (897.33, 1076.86)
**4**	742.06 (674.11, 810.02)	605.26 (553.37, 657.15)	730.64 (663.06, 798.22)	797.33 (718.69, 875.96)	836.39 (745.91, 926.86)	863.00 (771.06, 954.94)
**5**	658.06 (590.86, 725.27)	527.83 (479.52, 576.14)	638.16 (573.45, 702.87)	714.11 (635.16, 793.06)	761.65 (670.98, 852.31)	779.79 (687.04, 872.54)
**6**	592.11 (527.61, 656.61)	460.38 (416.31, 504.44)	562.55 (503.17, 621.94)	660.04 (580.70, 739.39)	696.04 (608.76, 783.33)	719.26 (625.53, 812.99)
**7**	548.69 (484.70, 612.68)	416.07 (374.15, 457.99)	514.39 (457.06, 571.73)	618.58 (539.27, 697.89)	653.62 (564.77, 742.46)	695.99 (599.99, 792)
**8**	504.08 (442.79, 565.36)	376.58 (337.31, 415.84)	469.39 (415.33, 523.45)	572.05 (496.04, 648.06)	614.40 (527.00, 701.81)	643.3 (549.03, 737.56)
**9**	466.91 (407.91, 525.91)	346.19 (309.26, 383.13)	429.56 (378.53, 480.59)	532.57 (458.95, 606.19)	574.17 (489.04, 659.31)	615.98 (520.03, 711.93)
**10**	434.91 (378.41, 491.42)	319.6 (285.19, 354.01)	398.59 (350.14, 447.05)	495.77 (426.17, 565.37)	540.66 (457.95, 623.37)	584.79 (489.00, 680.58)
**11**	407.90 (353.70, 462.10)	297.94 (265.39, 330.48)	373.73 (327.46, 420.01)	466.27 (399.59, 532.94)	509.33 (428.85, 589.81)	554.12 (459.31, 648.93)
**12**	384.41 (332.87, 435.96)	280.16 (249.23, 311.10)	351.36 (307.69, 395.03)	440.84 (377.15, 504.52)	482.41 (405.76, 559.05)	525.33 (432.24, 618.42)

© 2025 Rose L et al. *JAMA Network Open*.

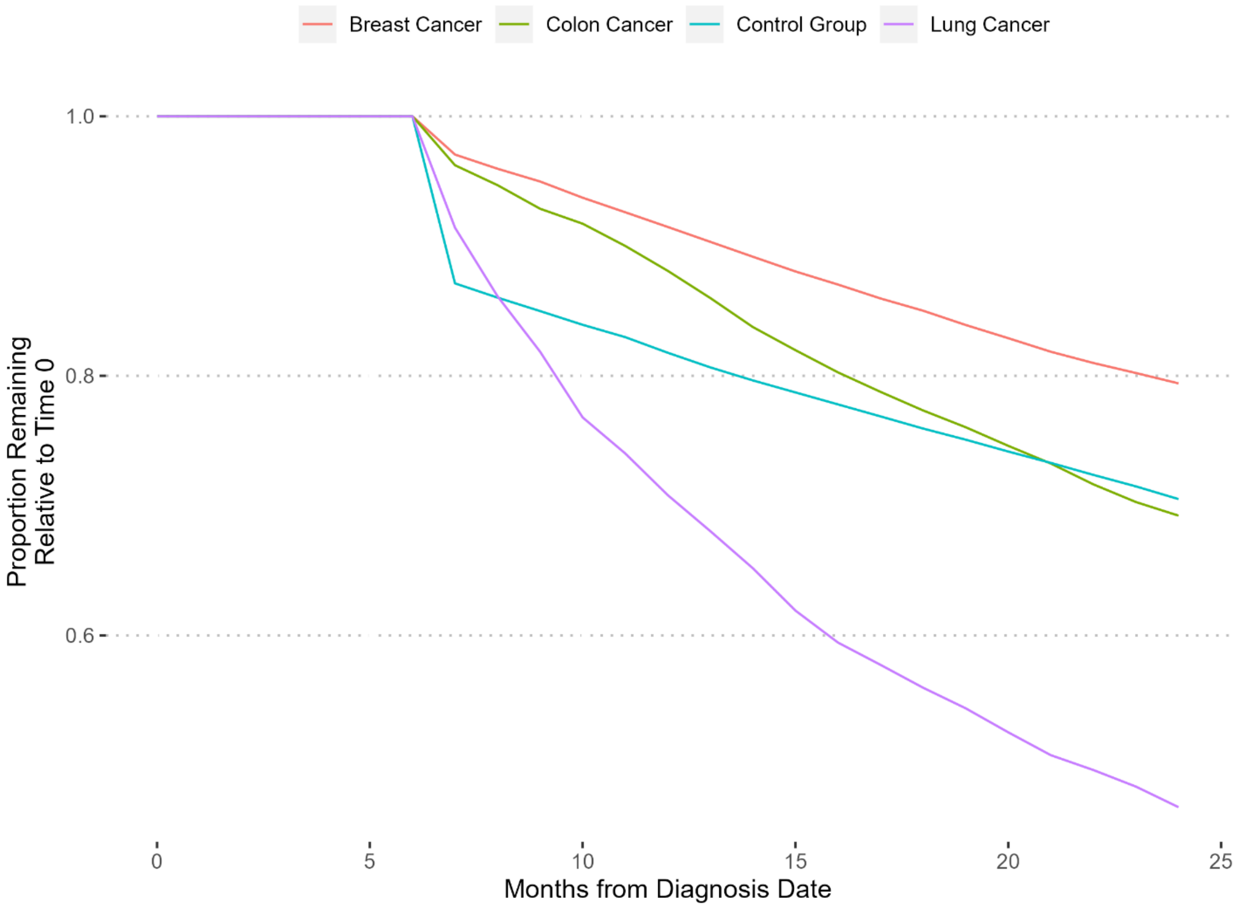

eFigure 1: Attrition from Sample by Cancer Status

© 2025 Rose L et al. *JAMA Network Open*.

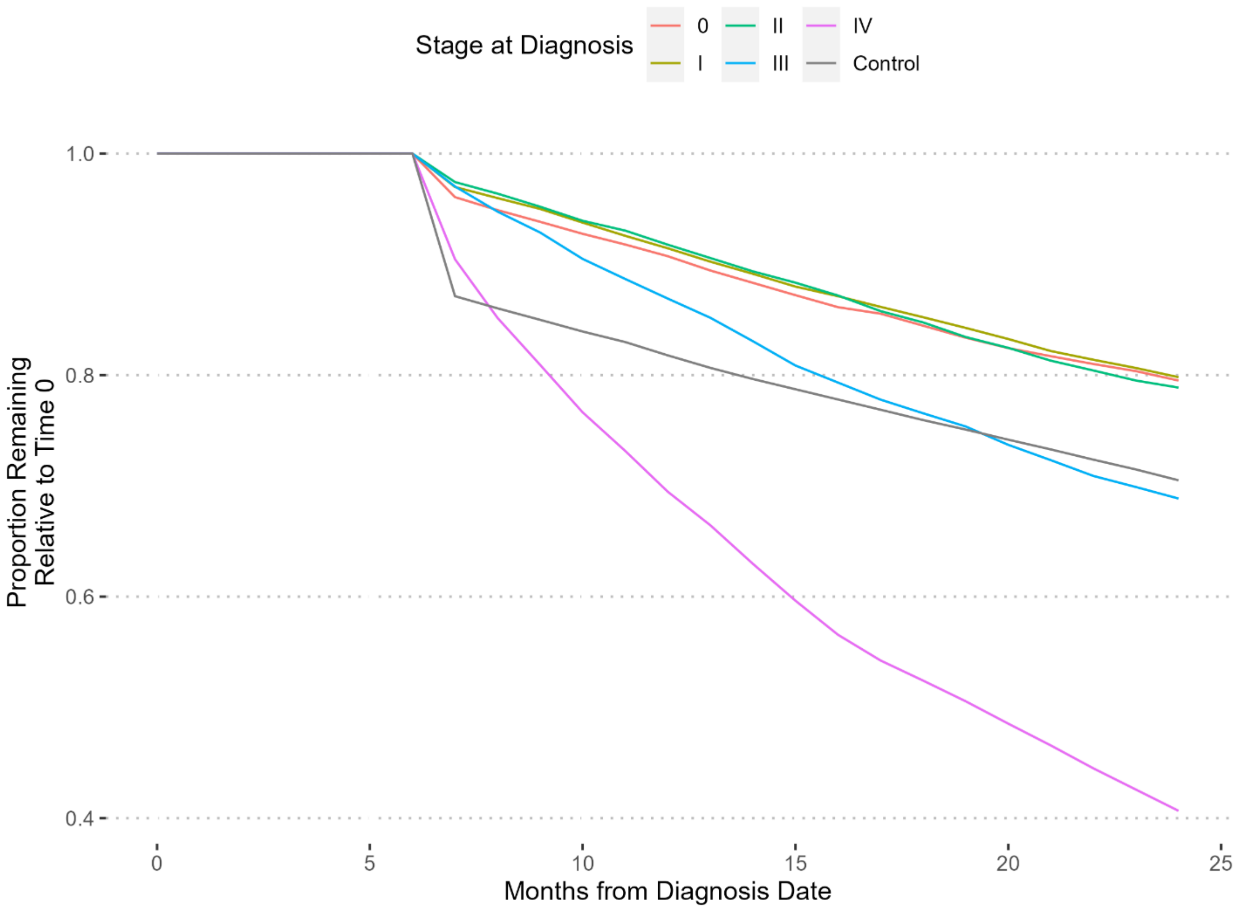

eFigure 2: Attrition from Sample by Stage at Diagnosis

© 2025 Rose L et al. *JAMA Network Open*.

eFigure 3a. Flowchart of Exclusions for Breast Cancer Patients

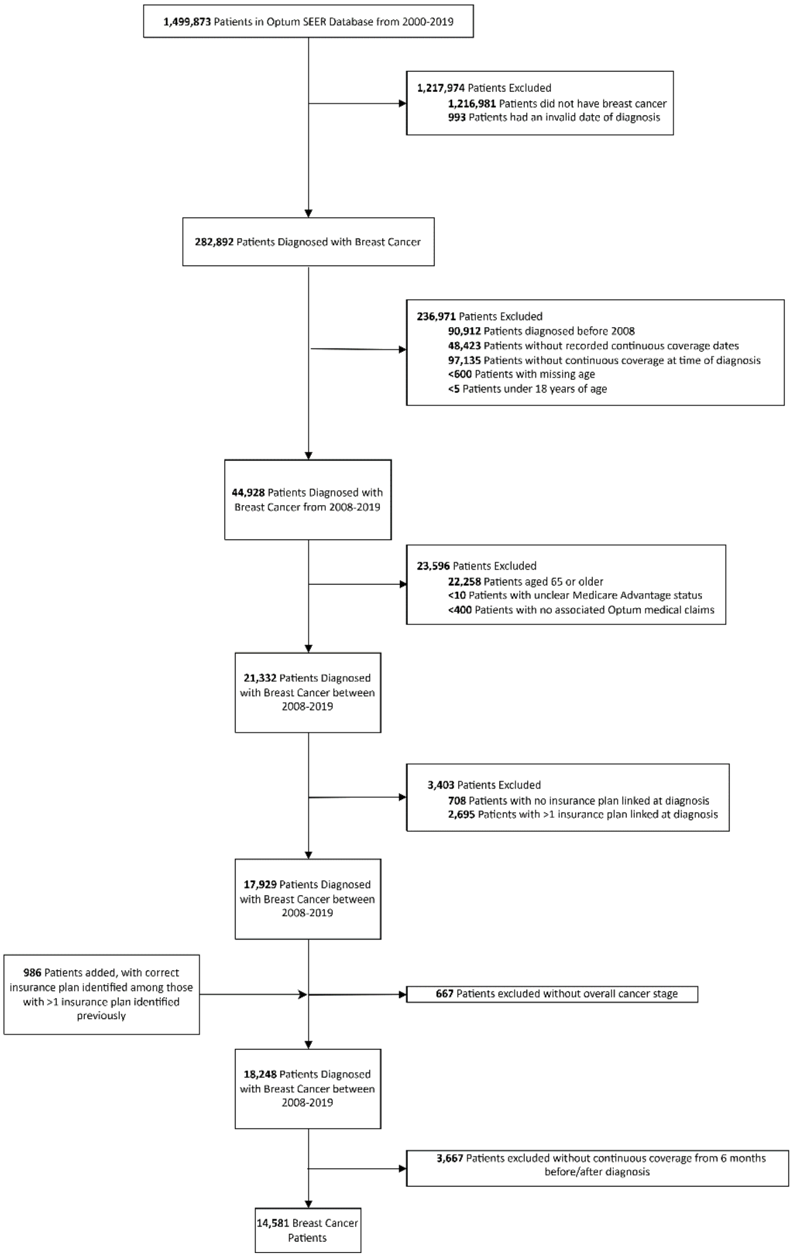

© 2025 Rose L et al. *JAMA Network Open*.

eFigure 3b. Flowchart of Exclusions for Lung Cancer Patients

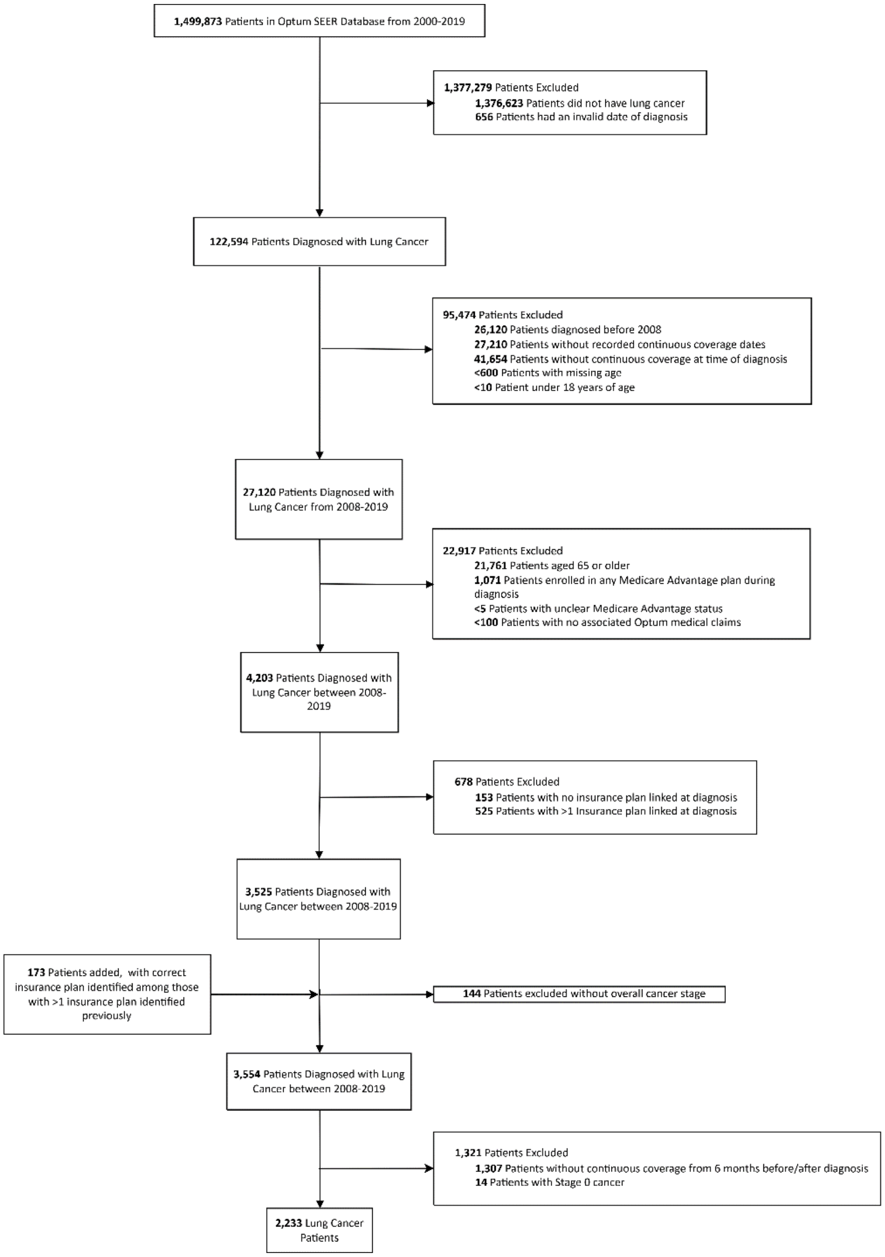

© 2025 Rose L et al. *JAMA Network Open*.

eFigure 3c. Flowchart of Exclusions for Colorectal Cancer Patients

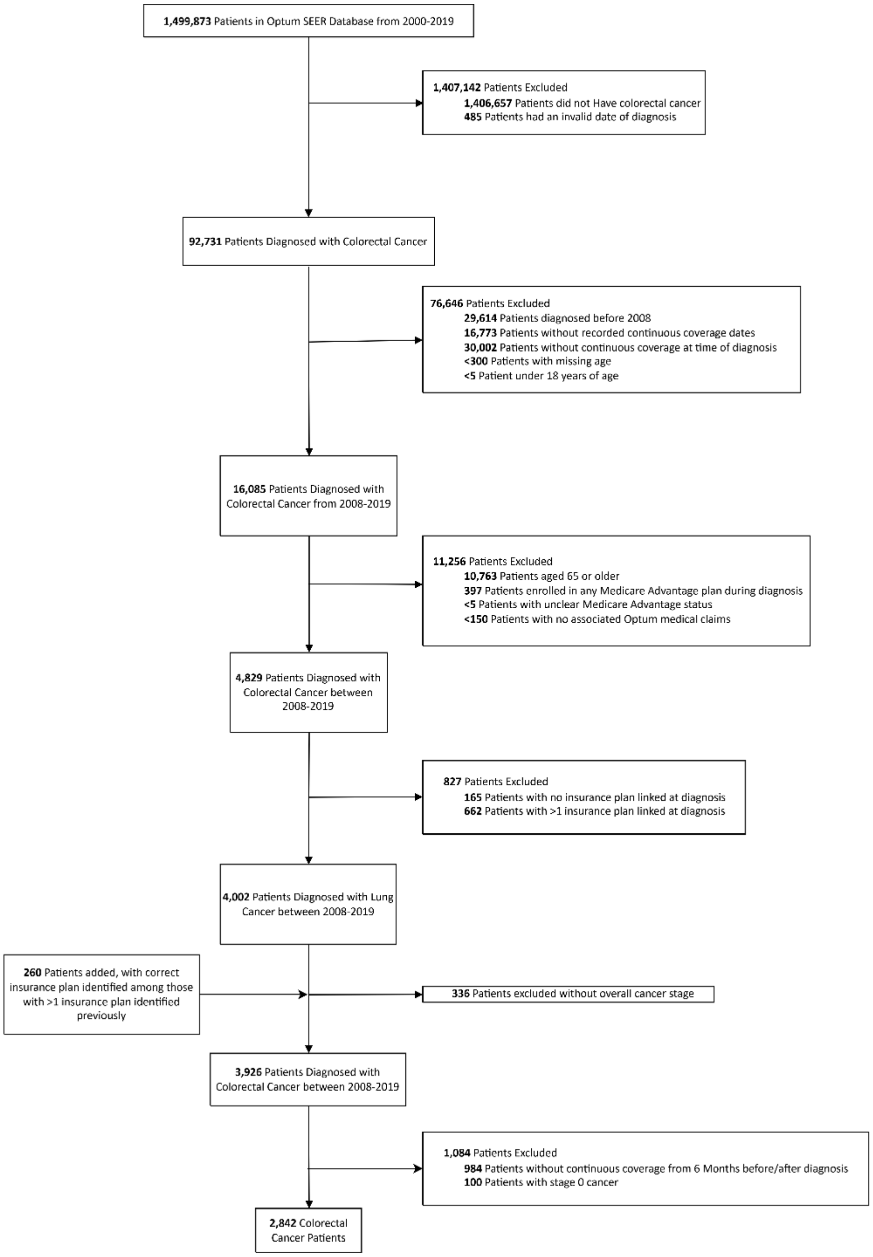

© 2025 Rose L et al. *JAMA Network Open*.

eFigure 3d. Flowchart of Exclusions for Control Group

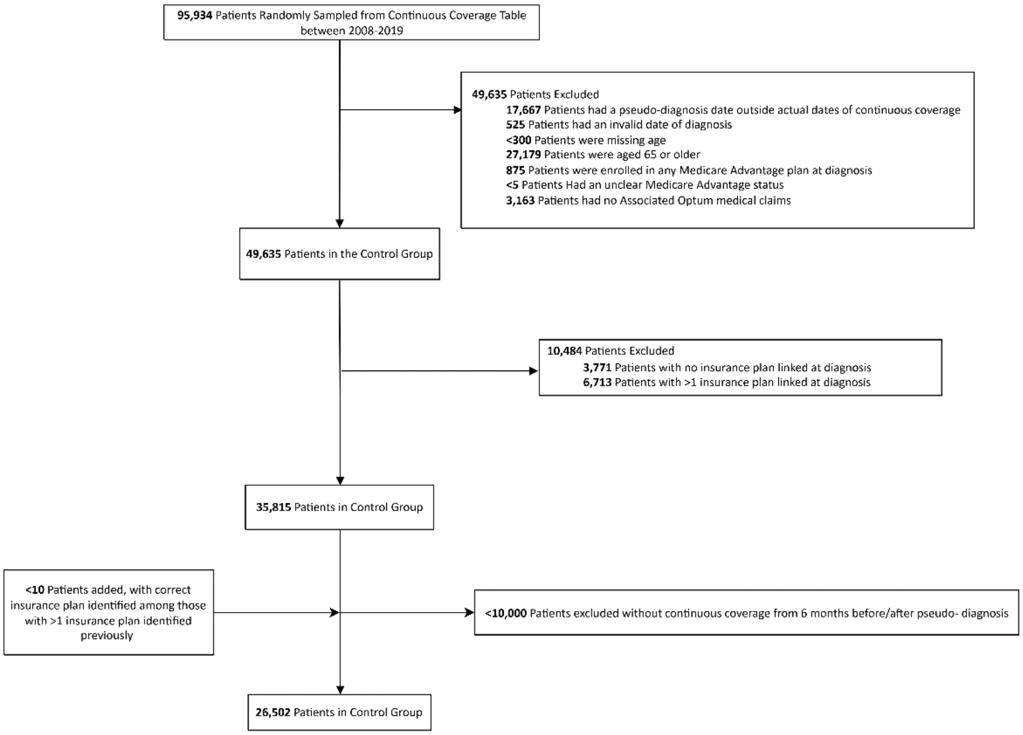

© 2025 Rose L et al. *JAMA Network Open*.
