# Supplementary material for: Estimated Out-of-Pocket Costs for Patients With Common Cancers and Private Insurance
Source: JAMA Netw Open. 2025 Jul 21;8(7):e2521575. doi: 10.1001/jamanetworkopen.2025.21575 (PMC12281234; doi:10.1001/jamanetworkopen.2025.21575)
Supplement: Supplement 2. — Data Sharing Statement [file jamanetwopen-e2521575-s002.pdf]

**Data Sharing Statement**

Rose. Estimated Out-of-Pocket Costs for Patients With Common Cancers and Private Insurance. *JAMA Netw Open*. Published online July 18, 2025. doi:10.1001/jamanetworkopen.2025.21575

**Data**
**Data available:** No
